# Revisiting Congenital Atransferrinemia: A Rare but Treatable Cause of Pediatric Heart Failure

**DOI:** 10.2174/011871529X399774251016072809

**Published:** 2025-11-10

**Authors:** Monika P. Jawanjal, Tom Devasia, Katta M Girisha, Shrikiran A. Hebbar, Prakashini K, Krishnananda Nayak

**Affiliations:** 1 Department of Cardiology, Kasturba Medical College, Manipal, Manipal Academy of Higher Education, Manipal, Karnataka, 576104, India;; 2 Department of Medical Genetics,Kasturba Medical College, Manipal, Manipal Academy of Higher Education, Manipal, Karnataka, India;; 3 Department of Pediatrics, Kasturba Medical College, Manipal, Manipal Academy of Higher Education, Manipal, Karnataka, India;; 4 Department of Radiodiagnosis and Imaging, Kasturba Medical College, Manipal, Manipal Academy of Higher Education, Manipal, Karnataka, India;; 5 Department of Cardiovascular Technology, Manipal College of Health Professions, Manipal Academy of Higher Education, Manipal, Karnataka, India

**Keywords:** Pediatric heart failure, iron overload cardiomyopathy, atransferrinemia, anemia, myocardial

## Abstract

Congenital atransferrinemia is an extremely rare autosomal recessive disorder characterized by functional or quantitative deficiency of transferrin. This leads to a characteristic clinical picture that includes heart failure due to iron overload cardiomyopathy, severe anemia, as well as hepatic and endocrine dysfunction in early infancy. Although rare or may be underdiagnosed, congenital atransferrinemia is a treatable cause of infantile heart failure and iron overload cardiomyopathy. Therefore, it is important to keep this diagnosis as a possibility in childhood presentation of heart failure and anemia. Early diagnosis and timely treatment can prevent progressive myocardial dysfunction and recurrent heart failure. This article focuses on pathophysiology, diagnosis, genetics, and management of heart failure in congenital atransferrinemia.

## 1 INTRODUCTION

First described by Heilmeyer *et al*. in 1961 in a 3-month-old girl with severe hypochromic anemia and iron overload, hereditary atransferrinemia is an exceptionally rare autosomal recessive disorder caused by functional or quantitative transferrin deficiency [1]. This condition disrupts iron delivery to erythroid cells, impairing hemoglobin synthesis, increasing iron absorption, and causing severe parenchymal iron overload.

Clinically, atransferrinemia presents with marked hypochromic microcytic anemia, infantile heart failure, and multiorgan endocrine dysfunction [1, 2]. Although it is a treatable cause of pediatric heart failure, it is often underdiagnosed due to a low index of suspicion and limited access to genetic testing. Fewer than 15 families worldwide have been documented with this condition, with consanguinity observed in two cases. The true prevalence of this rare entity remains unclear. Unrecognized cases may lead to catastrophic outcomes, particularly in young children. Progressive heart failure is the leading cause of death in these patients [3, 4].

Acquired atransferrinemia has also been reported in patients with liver or kidney disease, malignancies, and autoimmune or inflammatory disorders.

## 2 PATHOPHYSIOLOGY

The disorder stems from mutations in the *TF* gene on chromosome 3q21, which encodes transferrin, a critical iron-binding protein [5]. Transferrin regulates iron balance, provides antioxidative protection, and facilitates iron delivery to erythroid precursors [6, 7].

**Fig. (1):**
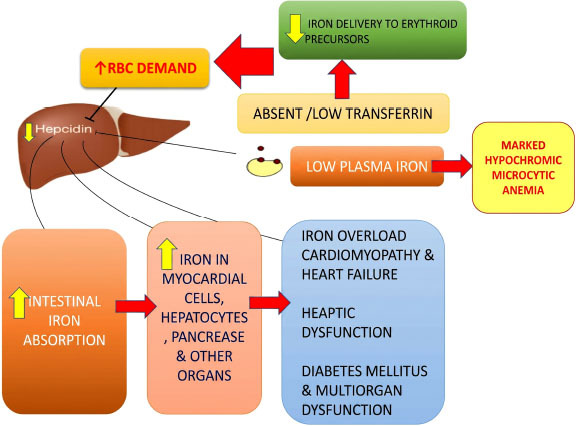
Mechanism of iron overload in atransferrinemia.

Its absence results in impaired hemoglobin synthesis, severe microcytic hypochromic anemia, suppressed hepcidin levels, and increased intestinal iron absorption [8]. The excess iron accumulates in parenchymal organs, such as the heart, liver, pancreas, and thyroid, leading to multisystem dysfunction and severe complications (Fig. **1**) [9]. Thus, early recognition and treatment are essential to prevent life-threatening outcomes in affected individuals.

## 3 MECHANISM OF IRON OVERLOAD IN THE HEART

In iron overload conditions, unbound ferrous iron (Fe^2+^) enters cardiomyocytes *via* voltage-gated L-type calcium channels. Iron deposition occurs first in the ventricular myocardium, followed by atrial tissue, and may also affect the conduction system. Myocardial iron uptake is slower than in hepatocytes, leading to delayed cardiac iron overload compared to the liver. Cardiomyocytes store iron in three forms: labile free iron, ferritin, and hemosiderin. Labile free iron, the most reactive form, generates reactive oxygen species (ROS) through the Fenton reaction, converting Fe^2+^ to ferric iron (Fe^3+^) and producing toxic hydroxyl radicals. These radicals cause lipid peroxidation, nucleic acid damage, and protein dysfunction, disrupting cellular antioxidant defenses. The resulting oxidative stress impairs calcium handling in cardiomyocytes, leading to defective excitation-contraction coupling, diastolic dysfunction, and eventually systolic dysfunction. Both the myocardium and epicardium are affected in iron-overload cardiomyopathies [10-12].

In atransferrinemia, non-transferrin-bound iron, the most bioactive form, accumulates early in life, causing free radical injury and antioxidant system disruption.

**Fig. (2):**
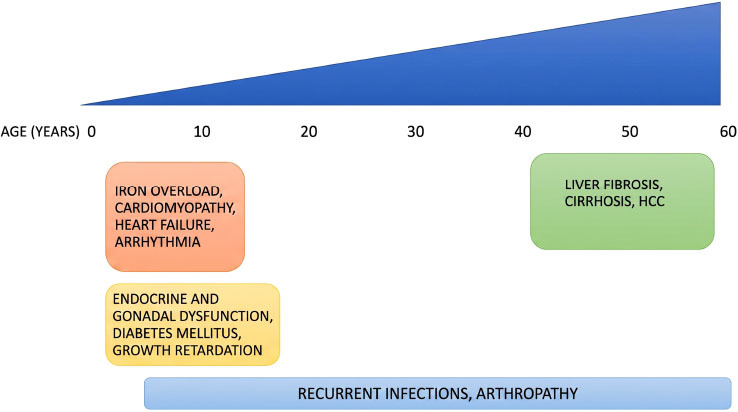
Age-wise evolution of iron overload in congenital iron overload disorders.

As per the natural history of congenital iron overload conditions, diabetes and heart failure occur in infancy or early childhood, and cirrhosis occurs later in life (Fig. **2**). However, no long-term data is available in patients with atransferrinemia. Hence, early recognition of this clinical entity is extremely important to prevent irreversible multiorgan dysfunction and death.

Other conditions leading to iron-overload cardiomyopathy, such as hereditary hemochromatosis, parenteral iron therapy, or hematological malignancies, follow similar mechanisms. Atransferrinemia may also manifest in conditions like nephrotic syndrome, GRACILE syndrome, and chronic alcoholism, which mandates the need for early detection to mitigate severe cardiac complications (Table **1**) [11].

## 4 CLINICAL MANIFESTATIONS OF HEREDITARY ATRANSFERRINEMIA

Hereditary atransferrinemia, characterized by extremely low or absent transferrin levels, affects major organs, including the heart, liver, and endocrine glands. Progressive heart failure remains the most common cause of death in reported cases [1, 2, 5].

It is important to understand when we should suspect atransferrinemia. The earliest presentation is in neonates with pallor and growth retardation due to severe hypochromic microcytic anemia. By infancy, myocardial iron overload combined with high-output circulatory states leads to heart failure. Endocrine dysfunction often emerges in infancy or early childhood, with diabetes mellitus and hypothyroidism further exacerbating heart failure and increasing mortality risk [2, 5].

Clinical findings include pallor, hepatomegaly, cardiomegaly, and signs of heart failure, such as dyspnoea, pedal oedema, and pulmonary rales. Laboratory investigations reveal severe anaemia, elevated serum ferritin, low serum iron, reduced TIBC, low transferrin saturation, and transferrin levels typically below 75 mg/dL (normal: 204-360 mg/dL) [1,4]. Peripheral smear shows microcytic hypochromic anaemia with anisopoikilocytosis, while bone marrow examination demonstrates erythroid hyperplasia and absent iron stores in later stages [5]. Despite anaemia, reticulocyte count is usually normal or low as there is no significant haemolysis [1, 13].

The combination of severe hypochromic microcytic anemia, elevated ferritin levels, hepatomegaly and heart failure, and diabetes with low transferrin saturation in infancy should raise suspicion of atrasnferrinemia. The diagnostic approach for the suspected case of atransferrinemia is elaborated in Fig. (**3**).

Diagnostic imaging reveals cardiomegaly on chest X-ray, sinus tachycardia, and left atrial enlargement on ECG. Echocardiography highlights early diastolic dysfunction through reduced global longitudinal strain (GLS) and abnormal myocardial performance indices (MPI). Progressive iron overload leads to systolic dysfunction, chamber dilation, and potential right ventricular dysfunction. Cardiac MRI confirms myocardial iron overload, and genetic testing identifies causative mutations, aiding diagnosis and genetic counselling [14].

Although most cases are diagnosed in infancy, delayed presentations can occur in late childhood with irreversible myocardial damage and refractory heart failure. Acquired forms of atransferrinemia, secondary to infection, liver disease, or malignancies, present similarly but may be identified later. Early detection through iron studies, cardiac imaging, and genetic testing is essential to initiate timely treatment and prevent severe complications.

## 5 CASE STUDY

**Fig. (4):**
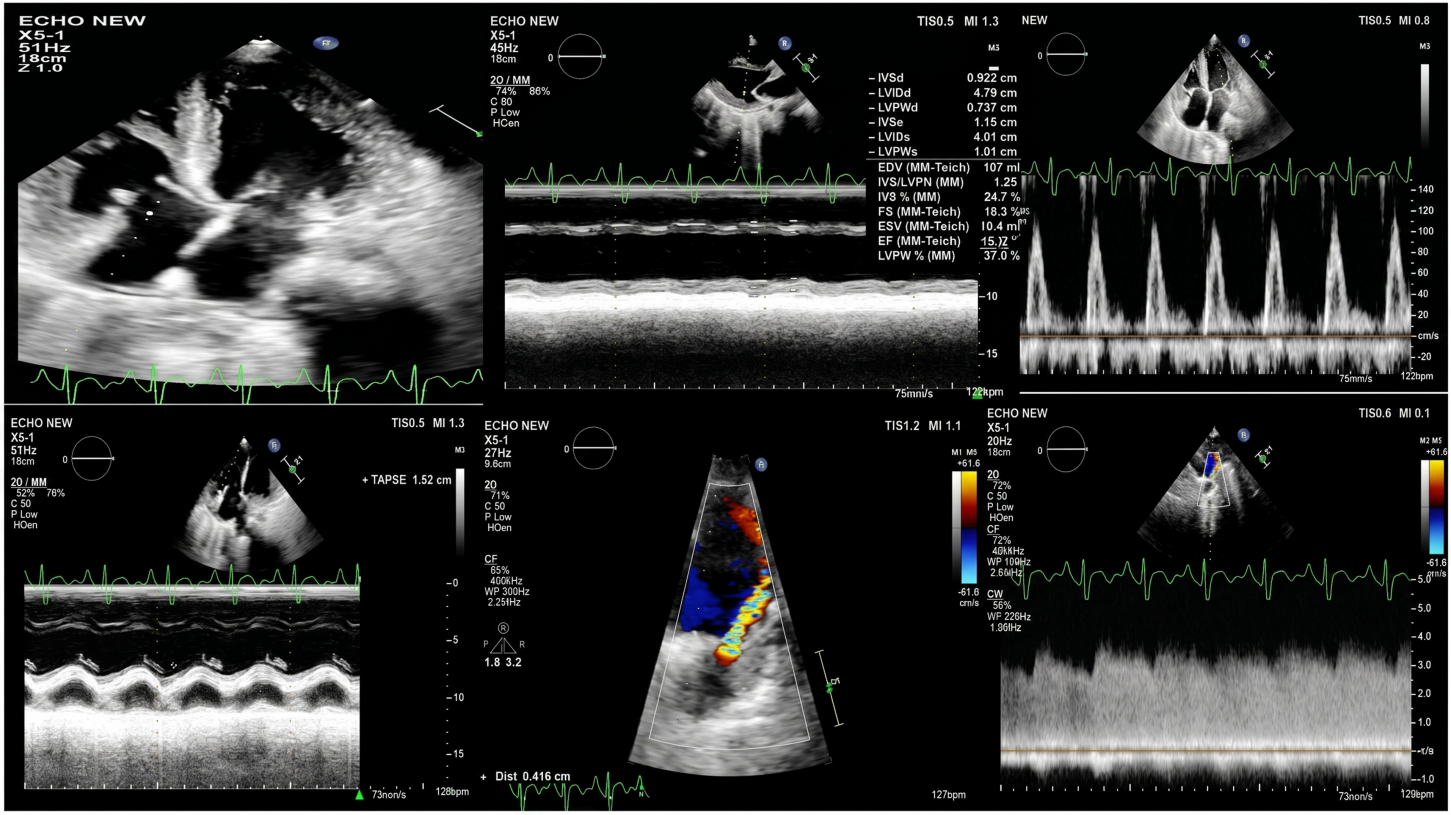
2D Echocardiogram of a child with atransferrinemia showing severe biventricular systolic dysfunction, LV diastolic dysfunction, and an incidental 4 mm-sized PDA.

A 13-year-old girl born of second-degree consanguinity presented with heart failure, severe anemia, and diabetes mellitus. Her clinical course included heart failure at six months, diabetes at age seven, and progressive LV systolic dysfunction. Iron studies revealed low TIBC, elevated ferritin, low transferrin saturation, and undetectable transferrin levels (<75 mg/dL). Echocardiography showed severe biventricular systolic dysfunction. There was an incidentally detected 4 mm PDA (Fig. **4**). As the age advanced, heart failure worsened disproportionately. Cardiac MRI confirmed myocardial iron overload, and genetic testing identified a novel mutation, confirming the diagnosis. This highlights the importance of early recognition and genetic analysis in hereditary atransferrinemia.

## 6 INVESTIGATIONS

Iron deposition in the heart occurs in a patchy distribution, rendering endomyocardial biopsies unreliable and no longer recommended. Liver iron concentration (LIC) can be measured noninvasively using a superconducting quantum interference device (SQUID), which is highly sensitive but limited by the availability of equipment [15].

Cardiac MRI with T2* imaging has become the gold standard for diagnosing and quantifying iron overload in the heart and liver, replacing liver biopsy [14, 16]. It enables early detection of cardiac siderosis, even before the onset of systolic dysfunction, using T2* relaxation parameters, which are inversely correlated with tissue iron levels. Reduced T2* signals indicate iron overload, and the modality also quantifies hepatic iron in the same session [17, 18].

Although hepatic iron levels normalize faster than cardiac iron (typically within six months), cardiac MRI provides a comprehensive assessment, including systolic and diastolic function, valvular regurgitation, and the extent of myocardial fibrosis or scarring. This information helps predict heart failure reversibility and guide tailored treatments, including iron chelation therapy [19, 20].

In atransferrinemia, early detection of cardiac and hepatic iron overload using CMR (Cardiac Magnetic Resonance) facilitates timely intervention, reducing mortality risk from heart failure. T2* imaging is highly sensitive and specific, offering critical insights for managing iron overload cardiomyopathies.

It remains indispensable for evaluating ventricular function, quantifying iron load, and modifying therapeutic approaches for reversible cardiomyopathies, including those caused by atransferrinemia [19, 20]. Key CMR features of iron overload cardiomyopathy are detailed in Table **2**.

## 7 CLINICAL CASE APPLICATION OF CARDIAC MRI IN IRON OVERLOAD

**Fig. (5):**
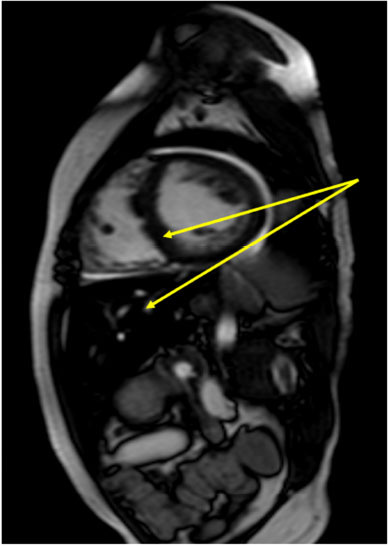
T2* Mid ventricular short axis acquisition in cardiac MRI showing that both the heart (Interventricular septum) and the liver have reduced and equal signal intensity (arrows), suggestive of severe iron overload.

**Fig. (6):**
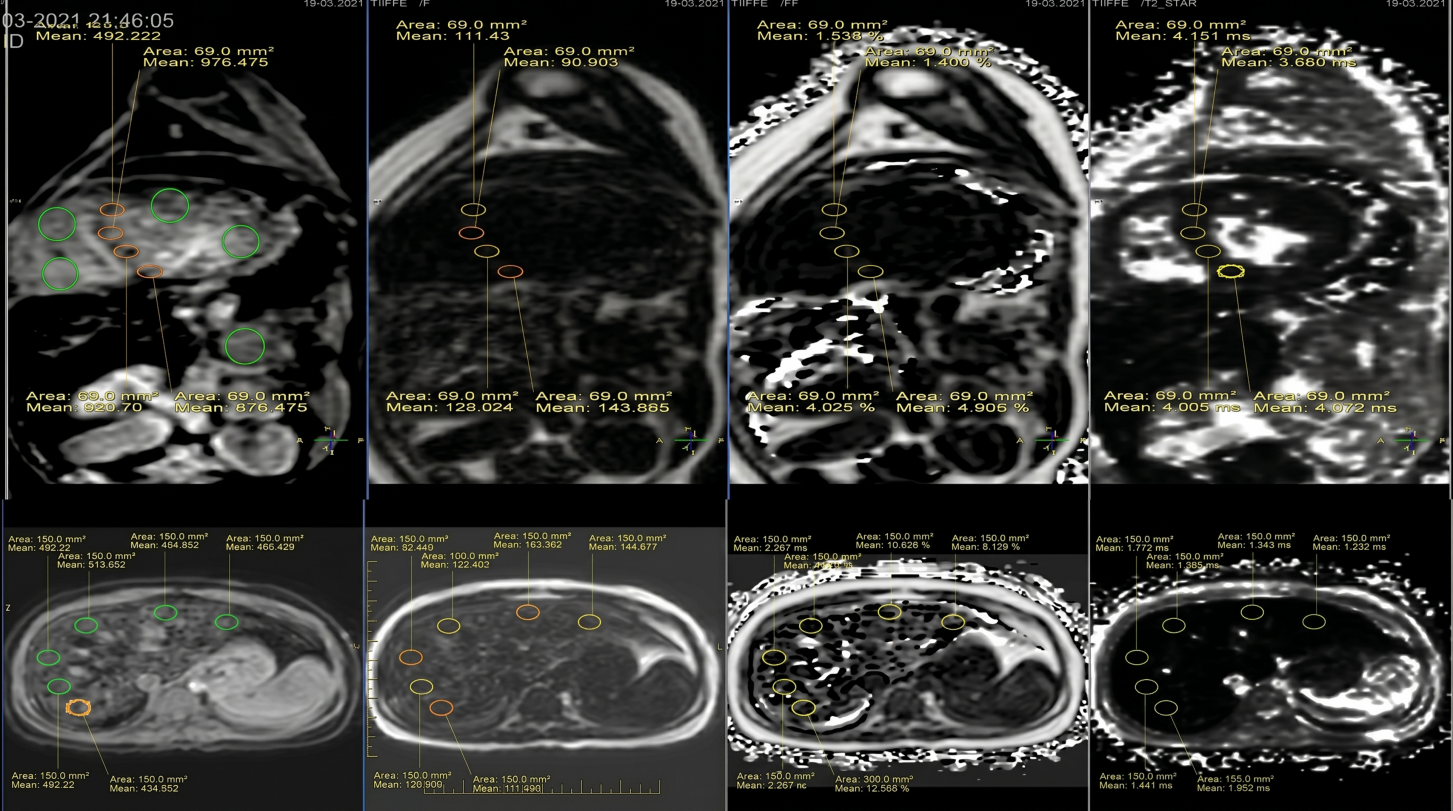
T2*MRI iron overload quantification, mean cardiac relaxation time was 4.7 and mean hepatic relaxation time was 1.4ms and mean cardiac iron concentration of 5.3mg per gram, and mean hepatic iron concentration of 17.8mg/g, suggestive of severe iron overload.

In the case discussed, cardiac MRI (CMR) revealed dilated cardiac chambers and severely depressed left ventricular (LV) systolic function with an ejection fraction (LVEF) of 24%, without Late Gadolinium Enhancement (LGE). T2* imaging in the midventricular short-axis acquisition showed significantly reduced and uniform signal intensity in the interventricular septum (IVS) and liver-a hallmark of iron overload cardiomyopathy (Figs. **5** and **6**).

T2* quantification estimated a cardiac iron concentration of 5.3 mg/g (normal <1 mg/g) and hepatic iron concentration of 17.8 mg/g (normal 0.8-1.16 mg/g), confirming severe iron overload. The diagnosis of iron overload cardiomyopathy was established through CMR before proceeding to genetic testing. The patient was initiated on iron chelation therapy with injectable desferrioxamine, followed by oral deferiprone. A repeat MRI was planned after six months to evaluate treatment efficacy and guide further management.

Cardiac MRI T2* remains a unique and reliable modality for diagnosing and quantifying cardiac iron overload and monitoring therapy in iron overload cardiomyopathies, including atransferrinemia. Its ability to detect early changes and guide treatment decisions makes it an indispensable tool in managing these conditions [19-21].

## 8 GENETIC TESTING

Genetic evaluation is an irreplaceable key to the definitive diagnosis of various cardiomyopathies and their risk stratification. Genetic testing plays a crucial role in the identification of the responsible genetic mutation in atransferrinemia as well. As this rare genetic disorder can be treated and the families of the affected child can be educated accordingly, genetic evaluation is essential in all cases of atransferrinemia.

As per Dabboubi *et al*., to date, around 18 cases have been reported worldwide, of which not all are genetically confirmed [1]. Also, they reported a novel homozygous deletion (c.293-63del) in intron 13 in a new case with congenital atransferrinemia in the year 2020. Beutler *et al*, who reported the first case of atransferrinemia in the United States and identified the TF gene mutation, postulated that atransferrinemia was reported in only 8 patients in 6 families till the year 2000 [22]. By 2014, fifteen families worldwide had been reported with transferrinemia, according to Nazir *et al*. [23]. So far, only two cases have been reported and genetically confirmed in India, both with a homozygous mutation in exon 13, as per Athiyarath *et al.* [24].

The exons along with exon-intron boundaries of the Transferrin gene are amplified with PCR using specific primers as previously described in the genetically evaluated cases of atransferrinemia [22]. In the above-mentioned patient, next-generation sequencing was performed using the Twist custom panel with Twist Biosciences.

**Fig. (7):**
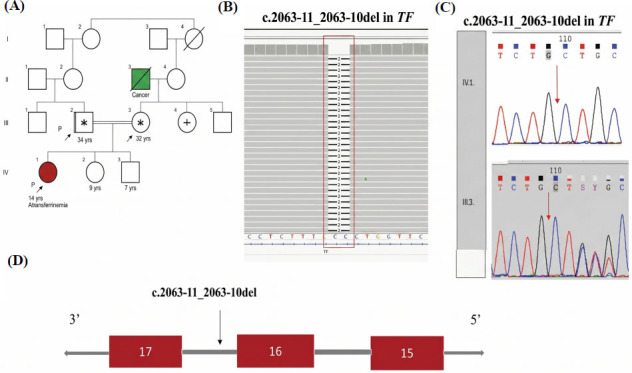
(**A**) Pedigree of the family; (**B**) Integrated genomic viewer of the variant c.2063-11_2063-10del) in intron 16 of *TF* in homozygous state. (**C**) Chromatogram from Sanger sequencing showing the variant c.2063-11_2063-10del) in homozygous state in proband and heterozygous state in mother; (**D**) Diagrammatic representation of *TF* variant with rectangle and straight line as exon and intron, and our variant located in intron 17 of *TF,* respectively.

Targeted exome sequencing in the proband revealed a novel splice site variant, NC_000003.12:g.133778575_133778576del(NM_001063.3:c.2063-11_2063-10del) in intron 16 of *TF* in the homozygous state. Segregation analysis in the mother confirmed the variant to be present in the heterozygous state. This variant was not observed in population databases and in-house whole-exome sequencing (WES) data of 1246 individuals of Indian origin (Fig **7**). This variant has been classified as a variant of uncertain significance according to the American College of Medical Genetics and Genomics (ACMG) guidelines.

The progression of heart failure in hereditary atransferrinemia and its correlation with specific genetic mutations remain unclear. Since iron overload cardiomyopathy is a treatable and reversible cause of recurrent heart failure in atransferrinemia, it is vital to consider this diagnosis in patients under age 20 years presenting with severe anemia and heart failure. Early evaluation with cardiac MRI and genetic testing is essential to prevent recurrent hospitalizations and mortality from progressive heart failure.

## 9 MANAGEMENT

The definitive treatment for atransferrinemia involves timely fresh frozen plasma transfusions, iron chelation, and intermittent phlebotomy [1, 25]. It is crucial to understand that iron therapy, often initiated for microcytic hypochromic anemia, can worsen iron overload in atransferrinemia if not identified.

The recommended therapy for treating iron overload in atransferrinemia includes monthly fresh frozen plasma transfusions to increase the transferrin pool and daily iron chelation therapy. Japanese studies have reported successful outcomes with monthly human apotransferrin infusions, although the high cost limits its accessibility [1, 25]. The standard foundational heart failure therapy with angiotensin receptor neprilysin inhibitors (ARNIs), sodium glucose cotransporter 2 inhibitors (SGLT2i), beta-blockers, and mineralocorticoid antagonists (MRA) can be useful. Diuretics can be used in acute heart failure for symptomatic relief. Although ARNIs and SGLT2 inhibitors have not been specifically studied in iron overload cardiomyopathies or atransferrinemia, they have shown efficacy in heart failure with reduced ejection fraction (HFrEF) in adults. The treatment should be tailored according to the clinical presentation of the patient [25].

Cardiac MRI plays a critical role in guiding treatment. If no myocardial fibrosis or scarring is present, iron chelation can significantly improve cardiac function. However, advanced cases with extensive fibrosis and refractory heart failure may require cardiac transplantation, although this is rarely performed in atransferrinemia due to risks of iron overload in the transplanted heart [26-30].

To ensure optimal outcomes, early and accurate diagnosis, coupled with lifelong treatment and meticulous follow-up, is essential. Monthly plasma infusions or purified apotransferrin therapy can help remove excess iron and restore transferrin levels, preventing myocardial damage and enabling effective hemoglobin synthesis. Genetic counseling is also vital for family planning, emphasizing the importance of reporting and managing this rare condition effectively.

## CONCLUSION

Hereditary atransferrinemia, a rare autosomal recessive disorder, typically presents in neonates or early childhood with severe hypochromic microcytic anemia, progressing to heart failure and diabetes mellitus. A combination of heart failure, severe anemia, and low transferrin saturation before adulthood is diagnostic. Although rare, its prevalence may be underestimated, warranting a high index of suspicion and thorough evaluation in pediatric patients presenting with anemia and heart failure. Moreover, treatment centers on meticulous heart failure management, periodic fresh frozen plasma transfusions, iron chelation, and phlebotomy. Early diagnosis and timely intervention significantly improve survival, although further data on long-term outcomes are needed.

As it is a review article limitations are not applicable. However there is limited data available about this rare entity, therefore this article may enhance the knowledge about congenital atransferrinemia and improve the clinical outcomes.

## Figures and Tables

**Fig. (3):**
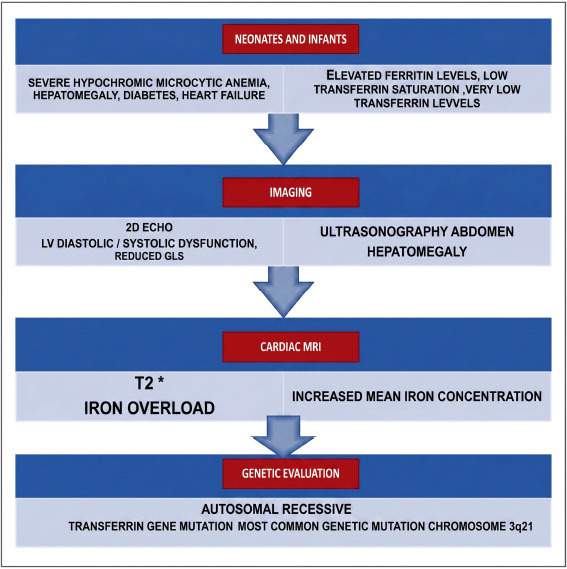
Diagnostic approach for the suspected case of atransferrinemia.

**Table 1:** Causes of iron overload cardiomyopathy*.

**Congenital**	**Acquired**	**Mscellaneous**
**JUVENILE ONSET**	Transfusion-related Fe overload (Thalassaemia, Sideroblastic anaemia)	African American iron overload
**HFE (6p21.3)**	Increased intestinal iron absorption is related to iron overload	Neonatal hemochromatosis
**TfR2 associated (7q22)**	Porphyria cutanea tarda	-
**FPN associated (2q32)**	Chronic hepatic disorders	-
**Atransferrinaemia (3q21)**	Alcoholic siderosis	-
**Acaeruloplasminaemia (3q23-24)**	Hepatitis B and C	-
**DMT1 deficiency (12q13)**	-	-
**Ferroportin disease (2q32)**	-	-
**Hereditary iron-loading anaemias with inefficient erythropoiesis**	-	-
**ADULT ONSET**	-	-
**HFE/TfR2 associated (7q22, 6p21.3)**	-	-
**HAMP associated (19q13)**	-	-
**HJV associated (1q21)**	-	-

**Note:** *[10].

**Table 2:** Characteristic MRI findings in atransferrinemia*.

**Characteristic MRI Features in Iron Overload Cardiomyopathy**	
1	In T2 imaging, the heart and liver show significantly reduced and similar signal intensity, considered as pathognomonic of iron overload cardiomyopathy.
2	CMR-derived T2* relaxation time is an important modality for quantifying cardiac iron overload, measured in a full-thickness area of the interventricular septum (IVS), and serves as a reliable indicator of global intramyocardial iron load.
3	T2* values are significantly reduced in iron overload, with a value less than 20 milliseconds highly suggestive of iron overload cardiomyopathy. (Normal >50milliseconds)
4	Increased mean iron concentration quantified using iron quantifying software (normal mean iron concentration in heart- < 1 mg / g (normal mean iron concentration in liver - 0.8-1.16 mg/g)
5	Ventricular systolic and diastolic dysfunction as well as valvular regurgitation
6	Late gadolinium enhancement (LGE) in presence of advanced disease with myocardial fibrosis/scarring

**Note:** *[16, 18, 19].

## LIST OF ABBREVIATIONS

ACEi: Angiotensin converting enzyme inhibitors
ARNI: Angiotensin receptor neprilysin inhibitor
AP4C: Apical four-chamber view
PSAX: Parasternal short axis view
DM: Diabetes mellitus
DMT1: Divalent metal transporter 1
ECHO: Echocardiography
FFP: Fresh frozen plasma
FPN: Ferroportin
GLS: Global longitudinal strain
GRACILE: Growth retardation, aminoaciduria, cholestasis, iron overload, lactic acidosis, and early death
HAMP: Hepcidin antimicrobial peptide
HFE: Human homeostatic iron (Fe++) regulator protein
HFrEF: Heart failure with reduced ejection fraction
HJV: Hemojuvelin
LGE: Late gadolinium enhancement
LIC: liver iron concentration
LV: Left ventricle
LVEF: left ventricular ejection fraction
MPI: myocardial performance index
MRI: Magnetic resonance imaging
PCR: polymerase chain reaction
PDA: Patent ductus arteriosus
RV: right ventricle
SGL2i: Sodium Glucose Cotransporter 2 Inhibitors
SQUID: superconducting quantum interference device
TAPSE: Tricuspid annular plain systolic excursion
TF: Transferrin
TfR2: Transferrin receptor 2
TIBC: Total iron binding capacity
